# Facial Paralysis Due to Brazilian Lyme Disease: A Case Report and Literature Review

**DOI:** 10.7759/cureus.48748

**Published:** 2023-11-13

**Authors:** Carlos E Levischi Jr

**Affiliations:** 1 Internal Medicine, Hospital Israelita Albert Einstein, São Paulo, BRA

**Keywords:** bell's palsy, lyme borreliosis, borrelia burgdorferi infection, facial paralysis, lyme disease

## Abstract

Lyme disease (LD) is a highly prevalent infection in the northern hemisphere, with an estimated incidence of 450,000 new annual cases in the United States and 65,000 new annual cases in Europe. Transmitted by the bite of a tick contaminated with a spirochete, the disease has three distinct stages. In the second phase of the disease, there may be neurological impairment, and the involvement of cranial nerve pairs occurs in this phase. Neuropathy of the seventh cranial nerve can occur in around 10% of adults with neurological involvement by Borrelia; in children, this incidence can reach 50%. A 32-year-old female patient presented with an abrupt facial paralysis that evolved into a difficult-to-resolve condition. With the appearance of probable contralateral involvement, LD was diagnosed. After the established treatment, the patient presented a good evolution of the symptoms.

## Introduction

Lyme disease (LD) was first recognized in an outbreak in the year 1975 in the community of Lyme, Connecticut in the USA, and affected patients who had arthritis and skin lesions, known as migratory erythema.

It is a non-contagious systemic infection caused by a spirochete bacterium of the genus *Borrelia* [1] and transmitted by the ticks of the *Ixodes ricinus* complex (in the Northern Hemisphere) and probably by the species *Amblyomma cajennense* (star tick) in Brazil [2], and by other more host-specific ticks, such as *Rhipicephalus sanguineus* (dog tick), *Rhipicephalus bovis* (cattle), and *Dermacentor nitens* (horse), which also bite humans [3].

In Brazil, the first cases were reported in 1987, and they were the first cases reported in Latin America [4].

LD has a cosmopolitan distribution and has been diagnosed on all continents. Described as endemic in regions of North America, Europe, and Asia, the disease is rarely reported in Brazil; however, cases are distributed across all regions of the country [5].

The tick has three stages of development. Soon after hatching from the egg, it is called a larva and has three pairs of legs. It needs to feed on blood to transform itself into a nymph, which already resembles an adult organism and has four pairs of legs. At that first moment, by sucking blood from a natural reservoir contaminated with *Borrelia*, the tick can already transmit the disease to humans. The nymph needs to feed on blood again, and from there, it matures into an adult. As natural reservoirs in our country, we have wild rodents, marsupials, and some species of birds, as well as cattle and horses [6].

The incubation period after the bite varies between three and 30 days, and as the body does not maintain natural immunity to the disease, a person can become reinfected from a new tick bite [7]. The disease usually manifests itself in three distinct clinical stages, which may vary according to the tissues or organs affected, the patient's immunity, and the duration of the infection [8].

The first phase, known as acute or localized disease, can occur between a few days and four weeks after the tick bite. At that moment, the patient may present a pathognomonic lesion of the disease, which is erythema migrans. In this phase, systemic symptoms of toxemia may also occur, such as fever, myalgia, arthralgia, headache, neck pain, pharyngitis, and lymphadenopathy. These symptoms will regress even without treatment.

The second phase, called the initial disseminated phase, can occur weeks to months after the tick bite. In this phase, cutaneous, cardiac, ophthalmological, and neurological involvement may occur. Approximately, 10% to 15% of patients may develop neurological symptoms in this phase. This involvement may include meningitis, encephalitis, both sensory and motor radiculoneuritis, and neuritis of the cranial nerves.

The third stage is called late disseminated disease or chronic disease. It happens months to years after the bite and can include various symptoms, such as arthritis, arthralgia, depression, and cognitive or even psychiatric changes, among many other symptoms.

Facial palsy in Lyme disease

Unilateral facial palsy is a fairly common diagnosis, with an incidence of 20-25 per 100,000 people. Most cases are linked to Bell's palsy, representing more than 50% of these cases [9,10]. In children with neurological involvement by LD, facial paralysis appears in approximately 50% of the cases [11-13]; this number drops to around 10% of the incidence of facial paralysis in adult patients with neuroborreliosis. Early diagnosis and institution of adequate treatment are extremely important for the prevention of serious complications.

The clinical presentation of facial paralysis associated with LD involves a rapid onset loss of muscle tone in all facial regions on the affected side, resulting in flaccid facial paralysis, with a gradual recovery in the following weeks, but full recovery can take up to months [14]. Even so, 16% to 23% of patients may have residual deficits and incomplete healing [15]. In LD, there may be bilateral facial paralysis. This finding is extremely unusual in other pathologies.

Clinically, this deficit is seen as permutations of hypo- and hyperactivity with undesirable coactivation of muscles, that is, synkinesis [16]. Such dysfunction results from aberrant neuronal regeneration, where axons extend to mimetic muscles other than those they should innervate [17,18]. It is not known why - despite appropriate treatment with antibiotic therapy - aberrant regeneration occurs in some patients after facial paralysis caused by LD, while other patients recover fully.

An important semantic discussion is necessary; facial paralysis is a symptom that may be associated with LD and is not Bell's palsy. Unfortunately, the replacement of the term Bell's palsy (which is a diagnosis) with facial paralysis that can occur as a symptom of LD occurs frequently in the literature [19-21]. Bell's palsy is a distinct mononeuropathy of the facial nerve, for which strong evidence points to viral reactivation in sensory neuron cell bodies within the geniculate ganglion [22] and the subsequent spread of viral infection to Schwann cells. The result is massive inflammatory infiltration of the nerve, resulting in edema and ischemic neural compression within the fallopian canal, subsequent Schwann cell death, and demyelination; in severe cases, permanent damage to the neural sheath occurs.

In contrast to Bell's palsy, direct infection of the facial nerve by *Borrelia* is unlikely to play an important pathophysiological role in facial paralysis in LD because the spirochete is rarely found in neural tissues [23-27]. The peripheral neuropathy of LD is a mononeuropathy multiplex with electrophysiology and biopsy findings consistent with axonal neuropathy [19,28,29]. Biopsies of peripheral nerves in humans and non-human primates with LD demonstrated multifocal Wallerian degeneration and axonal loss, with perivascular inflammatory infiltrates - more concentrated in the epineurium - characterized by a high percentage of lymphocytes and plasma cells, without evidence of necrotizing vasculitis or primary demyelination [19,23,24,27,29-32]. The inflammatory infiltrate in the absence of spirochete invasion that occurs in peripheral nerves in patients with LD points to autoimmunity as the underlying pathophysiology of facial paralysis in LD.

The pathophysiology of facial paralysis was studied in rats in a 2004 study by Eiffert et al. [33] and established that the particular vulnerability of human facial nerve injury may be the result of its long intraosseous path. This special anatomical aspect may be a predisposition for nerve inflammation to lead to injury due to high local pressure.

The first work that took into account a series of cases involving the seventh cranial nerve was published in 1985 by Steere et al., and it was a retrospective study with a total of 951 patients with LD [34]. Of these, 101 (10.6%) patients had facial paralysis caused by the disease. The incidence of facial nerve involvement in men and women was not statistically different, although bilateral involvement was more prevalent in males (74%). Facial paralysis affected both children and adults, ages ranged from three to 74 years, with an average of 34 years. In Steere's research, the involvement of the facial nerve was proportional between the right and left sides. In 38 of the 101 patients with facial paralysis, there were other neurological symptoms concomitantly. About 90% of patients had a complete recovery from paralysis with an average time of 26 days but with variations from one to 270 days for recovery.

In a paper published in 1999, Dotevall and Hagberg prospectively studied 120 patients admitted with a diagnosis of neuroborreliosis at a Swedish medical center [35]. In 38 (32%) patients, facial paralysis was one of the symptoms. Previous tick bite was recognized by 38% of patients and erythema migrans were reported by 34% of patients with facial paralysis. Most patients (86%) had pain or a distinct feeling of discomfort in the course of the nerve a few days before the onset of facial palsy, demonstrating the classic Garin-Bujadoux-Bannwarth syndrome. The syndrome is characterized by painful radiculopathy, neuropathy, varying degrees of motor weakness or facial nerve palsy, and lymphocytic pleocytosis of the cerebrospinal fluid. Bilateral paralysis occurred in 28% of patients, with some developing after antibiotic treatment was started. After six months of follow-up, 90% of the patients had completely recovered from their facial condition.

In a 2008 study, Nigrovic et al. reviewed the medical records of patients younger than 20 years who were admitted to the emergency room of a hospital located in the USA, in an area considered endemic for LD [36]. There were 106 patients with peripheral facial paralysis caused by the disease, 34% of all facial paralysis seen. The mean age of the affected children was 10.2 years, and there was no statistical difference between the most affected side (right x left). In this study, it was found that children who never had herpetic lesions during their lives, or children who had bilateral paralysis, had a greater chance that the cause of the paralysis was due to LD.

A more recent study, published in 2022 by Marques et al., found 44 patients with facial palsy in a total of 486 patients infected with LD [37]. Most of these patients were adults, with a mean age of 43 years. Only six of these patients had a past clinical history of tick bites. Facial paralysis was unilateral in 31 patients, with the right and left sides of the face affected in similar proportions. Thirteen patients had bilateral facial palsy, with 10 initially presenting with unilateral involvement, followed by contralateral facial nerve palsy a median of 6.5 days later (range 212 days). Interestingly, while unilateral facial paralysis occurred equally in men and women, bilateral paralysis occurred mainly in men (77% of patients with bilateral facial paralysis). Among all patients studied, 88% had complete recovery from the condition.

Other systemic symptoms of LD occur in up to 70% of patients with facial palsy due to neuroborreliosis [38] and may include fatigue, headache, arthralgia, neck pain, fever, or chills.

Regarding the prognosis of facial paralysis caused by LD, Bagger-Sjöbäck et al., in a 2005 study [39], followed 24 pediatric patients over a period of three to five years. Fifteen of these patients had some type of complaint of residual dysfunction in relation to the primarily affected side of the face. The patients underwent clinical and electrophysiological analyses regarding facial nerve function. Approximately half of patients with subjective symptoms of residual facial paralysis had mild signs of dysfunction on clinical examination. There was no correlation between the subjective feeling of facial dysfunction and the presence of signs of dysfunction on physical examination. Likewise, in about half of the subjective facial dysfunction group, as well as the control group (which did not present any facial symptoms), pathological values were found in neurophysiological examinations, through electroneuromyography.

## Case presentation

We present a 32-year-old female patient, born in and from the city of São Paulo, Brazil, living in an urban area, with a history of hypothyroidism controlled with Puran T4 25 mcg/day. She sought urgent medical attention on January 7, 2022, as she woke up in the morning with symptoms of numbness in her tongue and then loss of movement in the right hemifacial region. The symptoms had started a few days before, with a burning sensation on the lips, and as the patient had already presented a condition of labial herpes in the past, she believed it was a recurrence, but no local skin lesions appeared on the lips or other parts of the face.

She was examined by the clinical team at the emergency room and a right facial paralysis was detected, with a moderately severe degree of dysfunction and classification IV on the evaluation scale according to House and Brackmann, characterized by moderate paralysis, with muscle weakness, evident flaccidity, mimic inability to lift frontal muscle, incomplete eyelid closure, and asymmetrical mouth with maximum effort.

At the time of the consultation, the hypothesis of Bell's palsy was suggested, and prednisolone 20 mg per day was started for a period of five days. After three days, she underwent outpatient clinical care, where she did not report any improvement in her facial condition and even worse ophthalmological condition, with burning and redness in her right eye. At that moment, antiviral therapy was instituted with valaciclovir 1000 mg every eight hours for a period of seven days. The corticosteroid was also changed. The medication used at that time was prednisone, at a loading dose of 60 mg for another five days, with gradual reduction and total time of use for two weeks. During the consultation, methylcellulose eye drops were also prescribed during the waking period with guidance for palpebral occlusion and the use of retinol acetate ointment during sleep. On the sixth day of symptoms, physical therapy for facial muscles was started.

After 30 days of the initial symptoms, she had undergone 16 sessions of physiotherapy and reported perceiving a slight improvement in facial mobility, such as when smiling; however, she still had significant difficulty closing her right eyelid, and she maintained the ophthalmological guidelines previously passed. At that moment, it was recommended to initiate stimulation with acupuncture in an attempt to improve the condition. The points used during the acupuncture sessions were B62, LI4, ST4, ST5, ST6, ST7, LI20, ID18, VG20, and ID3.

**Figure 1:**
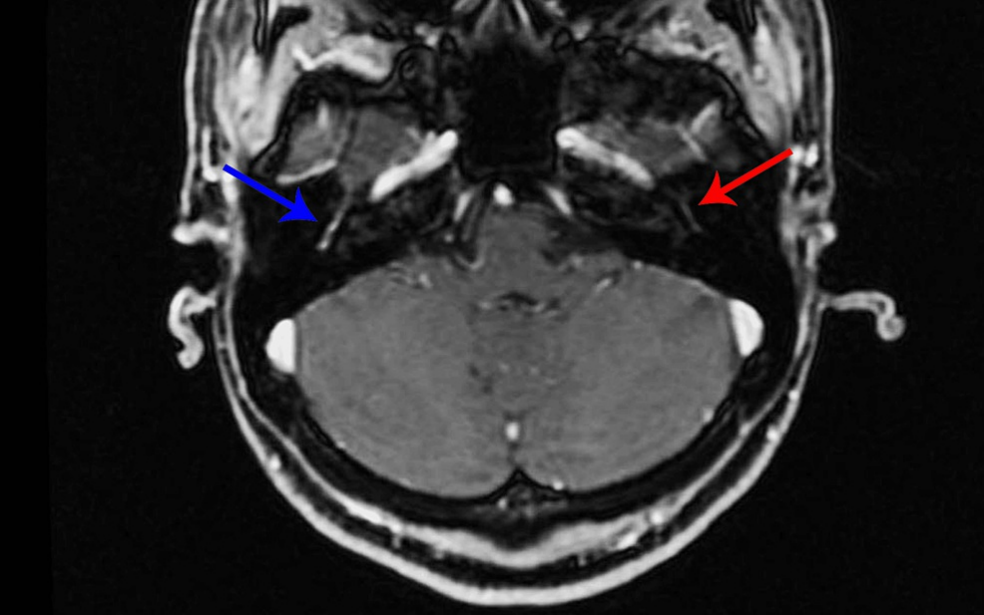
Contrast-enhanced axial MRI performed in February 2022 demonstrates abnormal enhancement of the right facial nerve (blue arrow). The left facial nerve (red arrow) is normal.

On February 15, 2022, she underwent a contrasted resonance imaging of the skull and face (Figure 1), and the only alteration found was described as “increased contrast and apparent regular thickening of the right facial nerve that extends from the fundic, labyrinthine, geniculate, and tympanic canalicular portion, to the posterior knee. Small infra styloid segment of the right facial nerve also appears to be thickened and contrasting.”

At the end of February, she underwent blood tests that showed no alterations in blood count and leukogram, as well as no changes in platelet count, erythrocyte sedimentation rate, and C-reactive protein. She did serology for herpes 1 and 2, cytomegalovirus, Epstein-Barr, rubella, SARS-CoV-2, and varicella-zoster; all showed positive IgG and negative IgM. Serology for toxoplasmosis was negative for both IgG and IgM. The venereal disease research laboratory (VDRL) test came with a negative result.

After 90 days of the initial condition, she still had difficulty closing the right eyelid completely, difficulty blinking, and paresis in the right hemifacial region. She was evaluated by a neurologist and suggested to maintain physical therapy and facial acupuncture. Physiotherapy frequency at that time was four times a week and acupuncture once a week.

At 120 days, she started to complain of sparse spasms and involuntary movements that occurred with the movement of a different facial muscle group (synkinesis) and accompanied by some pain when moving the right side of the face. At that time, she still had lip rhyme deviation and incomplete right eye closure, but there was a significant improvement in relation to the onset of symptoms.

In August 2022, she complained of fatigue, which she attributed to stress and anxiety. She had esthetic concerns due to the mimic musculature still maintaining a slight alteration in relation to the contralateral side. She also reported feelings of weakness and tingling in the upper limbs, as well as headaches after the end of the daily workday. She began to reduce the number of weekly physical therapy sessions for her face and maintained acupuncture sessions every two weeks.

In October 2022, her complaints regarding her face were difficulty keeping her mouth closed without effort, with air escaping at times, sporadic facial spasms, and esthetic dissatisfaction.

**Figure 2:**
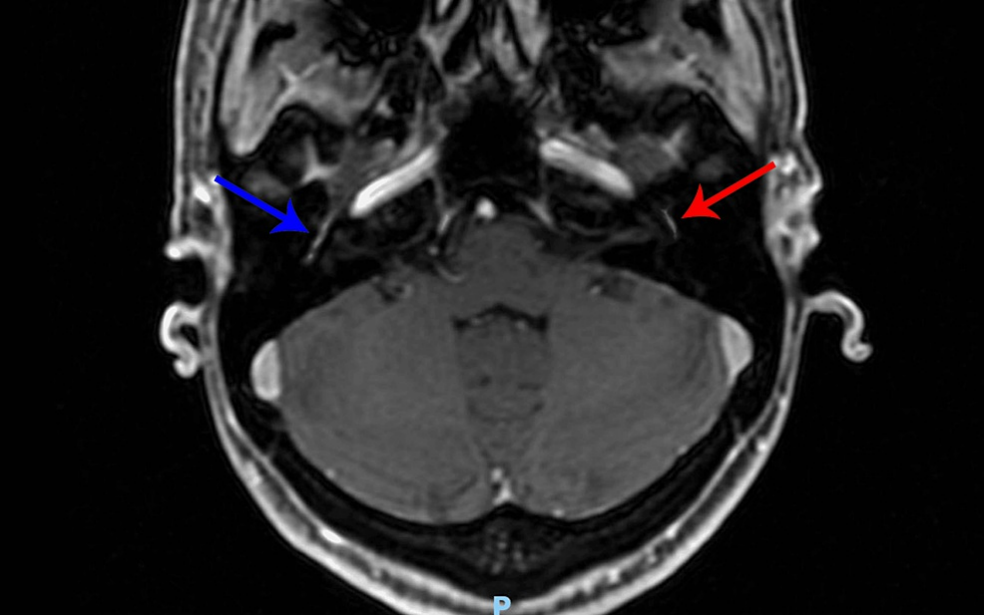
Contrast-enhanced axial MRI performed in October 2022 demonstrates abnormal enhancement of the right facial nerve (blue arrow) and left facial nerve (red arrow).

On October 28, 2022, the skull and face MRI with contrast was repeated (Figure 2) and the only alterations were described as follows: “increased contrast and apparent regular thickening of the bilateral facial nerve, more evident on the right, which affects the labyrinthine, geniculate, and tympanic.”

Given the findings detected on the MRI, even though the patient did not present symptoms related to the left side of the face, serology for LD was requested. The test showed positivity in the IgM serology performed by the enzyme-linked immunosorbent assay (ELISA) method and in the supporting western blot, the antibody against the 23 Kd protein was positive. In both exams, the IgG was negative for the pathology.

Taking into account the patient's clinical and radiological picture, as well as her slow evolution, it was decided to perform the treatment of LD. The option made was the use of ceftriaxone 2 g a day for 28 days. The scheme was carried out through the insertion of a peripherally inserted central catheter (PICC) with infusions performed once a day by the homecare team. The therapy continued for the stipulated period without any complications. The patient was monitored and did not present changes in liver or kidney function during the infusion period or in the subsequent weeks.

The patient reported improvement in the spasms and the esthetic appearance of the right hemifacial region, as well as the general picture of fatigue, headache, and tingling.

**Figure 3:**
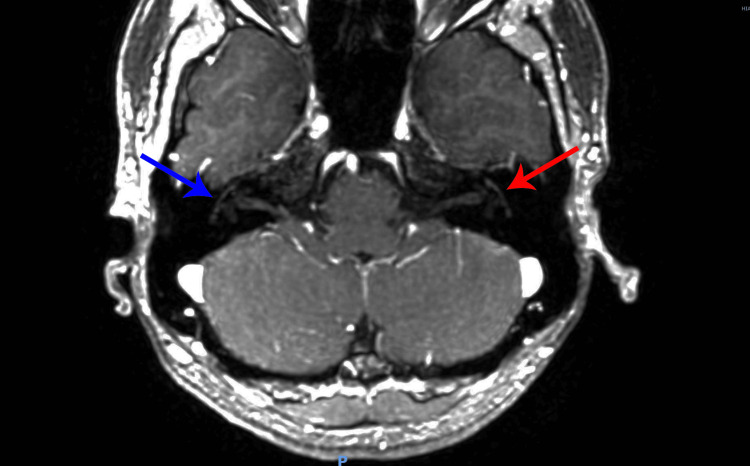
Contrast-enhanced axial MRI performed in February 2023 demonstrates normal right facial nerve (blue arrow) and normal left facial nerve (red arrow).

Thirty days after the end of the antibiotic therapy, a new MRI was performed on February 13, 2023 (Figure 3), which showed the involution of the inflammatory activity in both facial nerves. The report states “slight asymmetry with possible thickening of the geniculate ganglion on the right, but there were no areas of anomalous or asymmetrical enhancement along the path of the facial nerves. The findings are nonspecific and may correspond to previous partially involuted inflammatory alterations without activity in the present exam or correspond to the constitutional variant. Correlate with clinical data.”

At the moment, she maintains control of the residual symptoms of synkinesis by using botulinum toxin in a satisfactory manner.

## Discussion

Clinical history and physical examination are usually enough to make the diagnosis of facial palsy, and imaging methods are usually unnecessary. However, they are well used in cases where there are atypical symptoms, including slow or incomplete recovery or even recurrence [40]. Regarding the imaging exam of choice, it may depend on the clinical symptoms, as well as on the availability of each method [41]. Specifically, MRI with gadolinium is the modality of choice for lesions located in the parotid gland, pontine cerebellar angle, and internal auditory canal, while high-resolution CT is preferred for pathologies of the temporal bone [42,43].

Although the patient in the case in question did not present symptoms of bilateral facial paralysis, her imaging examination showed bilateral involvement of the seventh cranial nerve. Bilateral paralysis should draw attention to systemic disturbances, unlike unilateral paralysis, which has a greater connection with local factors [44]. The etiologies of bilateral facial palsy may include congenital, infectious, vascular, metabolic, autoimmune, neoplastic, neurological, or traumatic causes [45,46]. It is crucial to identify the underlying cause because treatment and prognosis depend on it. LD is the most common among other infectious causes of bilateral facial nerve paralysis and accounts for 30% to 35% of cases [47].

Diagnosis of LD should be made clinically in patients who present with erythema migrans. Do not rule out the possibility of LD in people with symptoms but no clear history of tick exposure. Serological testing for antibodies is only recommended to support clinical suspicion in patients with symptoms or signs consistent with LD. Serology is recommended to be performed in two steps, and an enzyme immunoassay (ELISA) can be performed as the first step; if positive or indeterminate, it must be followed by a confirmatory western blot, which requires correct interpretation of positive bands [48]. For the diagnosis of neuroborreliosis, the clinical picture combined with the serology collected from the blood is usually enough to make the diagnosis of the neurological involvement caused by LD, and the collection of cerebrospinal fluid (CSF) is not necessary unless the objective is to rule out other pathologies [49]. Corroborating this information, there are no clear criteria for diagnosing the pattern of bands detected when the western blot is performed on the CSF, such as the criteria established for the diagnosis on blood [50].

All manifestations of borreliosis need to be treated with antibiotics. The type of antibiotic applied, and the duration of treatment will depend on the stage and severity of the disease. Erythema migrans, cutaneous lymphocytoma, Lyme arthritis, and chronic atrophic acrodermatitis are mainly treated orally. If there are neurological symptoms, severe cardiac involvement, or ocular manifestations, intravenous treatment is initially recommended. For oral therapy, doxycycline, amoxicillin, cefuroxime, and, if intolerance is demonstrated, azithromycin is available. For intravenous treatment, ceftriaxone, cefotaxime, or penicillin G is used. Benzathine penicillin can be an intramuscular form of treatment. Except for migratory erythema, all manifestations must be subjected to a careful diagnostic investigation before starting treatment [51].

Regarding the best therapy to be instituted in these patients, antibiotic therapy is certainly a necessary path, but the use of corticosteroids in cases of facial paralysis due to LD is something that is still under discussion, and its use is indicated with level of evidence 1a for cases of facial paralysis of viral etiology [52,53]. Reducing residual dysfunction caused by aberrant regeneration of the facial nerve, which leads to deficits in function, as in synkinesis [54].

In a retrospective study published in 2017, Jowett et al. studied 51 patients with facial paralysis due to LD [55]. These patients were divided into three groups, where 18 (26.5%) patients received antibiotics only, 17 (25%) received antibiotics and corticosteroids, and 16 (23.5%) patients received triple therapy with antibiotics, corticosteroids, and antivirals. Patients were followed for an average of 15.1 months (three weeks to 84 months) and a significant sequelae deficit was found in patients in the corticosteroid groups (p = 0.031), more pronounced in patients who were followed for more than 12 months. The authors concluded that the use of corticosteroids in patients with facial paralysis due to LD leads to worse results of facial neuromuscular function in the long term.

In a prospective study published in 2018, Wormser et al. followed 14 patients with facial paralysis due to LD for 12 months. Of these, 11 (78.6%) received both antibiotics and corticosteroids [56]. A total of six (54.5%) patients in this group had some degree of sequelae dysfunction at the assessment performed 12 months after diagnosis (95% CI: 28% to 78.7%). Dysfunction was defined by residual facial muscle weakness. It was also defined by the presence of synkinesis, tension, and discomfort in the previously affected face region, hypertonicity of the facial muscles, restriction of facial movements, and/or gustatory epiphora (Bogorad syndrome). These sequelae are called post-facial palsy dysfunction [57] or even post-facial palsy syndrome [58]. The authors acknowledge that this is a small group of patients and that further studies on the topic are needed to determine the risk-benefit ratio of using corticosteroids in patients with facial paralysis caused by LD.

Historically, choosing the best technique for facial rehabilitation has been hampered by the lack of scientific evidence supporting specific therapies. The literature review between 1958 and 2001 reveals only three randomized controlled trials [59]. This led to many cases of facial paralysis being advised to wait spontaneously for the condition to improve. However, the number of publications with case series that demonstrate faster improvement of patients with the use of facial rehabilitation techniques through physiotherapy, biofeedback, and local massage techniques has been growing [60].

Acupuncture is a low-risk and safe therapeutic method for several diseases, and there is no evidence of deleterious effects from the use of this therapy in cases of facial paralysis [61]. It can be an effective method in an attempt to reduce the incidence of sequelae and improve symmetry [62].

Certainly, synkinesis is one of the most feared complications in patients with post-facial palsy sequelae. It can cause functional limitation, causing difficulties in performing basic activities, such as eating, drinking, smiling, or even blinking the eyes [63]. The most modern therapeutic modalities for the treatment of facial synkinesis include the use of botulinum toxin type A (BT-A) with injections for selective chemodenervation of the affected muscle groups and facial neuromuscular retraining. Other treatment options include surgical therapies such as selective neurolysis or myectomy, although these were almost rendered obsolete with the advent of BT-A [64].

## Conclusions

LD is clinically complex, especially when we are dealing with patients in the disseminated stages of the infection. Remember that patients can present in the second or third stages of the disease even without having a clinical history of the symptoms of the previous stages. It is imperative that emergency room physicians and otolaryngologists should consider the diagnosis of LD when caring for patients with a history of facial paralysis.
